# Creatininium cinnamate

**DOI:** 10.1107/S1600536811016916

**Published:** 2011-05-11

**Authors:** A. Jahubar Ali, S. Athimoolam, S. Asath Bahadur

**Affiliations:** a Department of Science and Humanities, National College of Engineering, Maruthakulam, Tirunelveli 627 151, India; b Department of Physics, University College of Engineering Nagercoil, Anna University of Technology Tirunelveli, Nagercoil 629 004, India; c Department of Physics, Kalasalingam University, Anand Nagar, Krishnan Koil 626 190, India

## Abstract

The crystal structure of the title compound (systematic name: 2-amino-1-methyl-4-oxo-4,5-dihydro-1*H*-imidazol-3-ium 3-phenyl­prop-2-enoate), C_4_H_8_N_3_O^+^·C_9_H_7_O_2_^−^, is stabilized by N—H⋯O hydrogen bonding. Cations are linked to anions to form ion pairs with an *R*_2_^2^(8) ring motif. These ion pairs are connected through a *C*_2_^2^(6) chain motif extending along the *c* axis of the unit cell. This crystal packing is characterized by hydro­phobic layers at *x* ∼ 1/2 packed between hydro­philic layers at *x* ∼ 0.

## Related literature

For related structures, see: Bahadur, Kannan *et al.* (2007 ▶); Bahadur, Sivapragasam *et al.* (2007 ▶); Bahadur, Rajalakshmi *et al.* (2007 ▶). For hydrogen-bonding motif notation, see: Bernstein *et al.* (1995 ▶). For crystal engineering, see: Desiraju (1989 ▶). For information about creatinine and its biological significance, see: Madaras & Buck (1996 ▶); Sharma *et al.* (2004 ▶); Narayanan & Appleton (1980 ▶). 
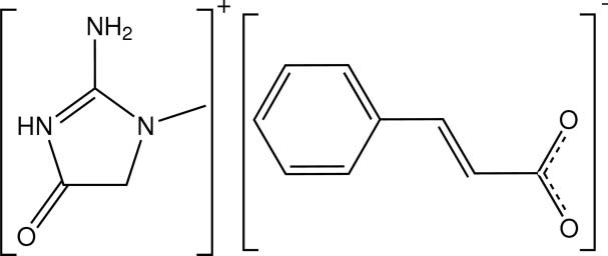


## Experimental

### 

#### Crystal data

C_4_H_8_N_3_O^+^·C_9_H_7_O_2_^−^
*M* *_r_* = 261.28
Monoclinic, 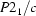

*a* = 9.1680 (8) Å
*b* = 11.3391 (11) Å
*c* = 12.7070 (12) Å
β = 104.578 (2)°
*V* = 1278.5 (2) Å^3^
*Z* = 4
Mo *K*α radiation
μ = 0.10 mm^−1^
*T* = 293 K
0.25 × 0.22 × 0.18 mm

#### Data collection

Bruker SMART APEX CCD area-detector diffractometer
9014 measured reflections
2250 independent reflections
2037 reflections with *I* > 2σ(*I*)
*R*_int_ = 0.023

#### Refinement

*R*[*F* ^2^ > 2σ(*F* ^2^)] = 0.035
*wR*(*F* ^2^) = 0.100
*S* = 1.05
2250 reflections
186 parameters
H atoms treated by a mixture of independent and constrained refinement
Δρ_max_ = 0.16 e Å^−3^
Δρ_min_ = −0.14 e Å^−3^

### 

Data collection: *SMART* (Bruker, 2001 ▶); cell refinement: *SAINT* (Bruker, 2001 ▶); data reduction: *SAINT*; program(s) used to solve structure: *SHELXTL/PC* (Sheldrick, 2008 ▶); program(s) used to refine structure: *SHELXTL/PC*; molecular graphics: *Mercury* (Macrae *et al.*, 2008 ▶) and *PLATON* (Spek, 2009 ▶); software used to prepare material for publication: *SHELXTL/PC*.

## Supplementary Material

Crystal structure: contains datablocks global, I. DOI: 10.1107/S1600536811016916/bt5542sup1.cif

Structure factors: contains datablocks I. DOI: 10.1107/S1600536811016916/bt5542Isup2.hkl

Supplementary material file. DOI: 10.1107/S1600536811016916/bt5542Isup3.cml

Additional supplementary materials:  crystallographic information; 3D view; checkCIF report

## Figures and Tables

**Table 1:** Hydrogen-bond geometry (Å, °)

*D*—H⋯*A*	*D*—H	H⋯*A*	*D*⋯*A*	*D*—H⋯*A*
N4—H4⋯O11^i^	0.899 (18)	1.840 (18)	2.7373 (15)	177 (2)
N5—H5*A*⋯O11^ii^	0.899 (18)	1.959 (18)	2.8403 (16)	166 (1)
N5—H5*B*⋯O12^i^	0.929 (19)	1.754 (19)	2.6663 (16)	167 (2)

Symmetry codes: (i) 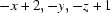; (ii) 
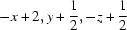
.

**Fig. 1.**
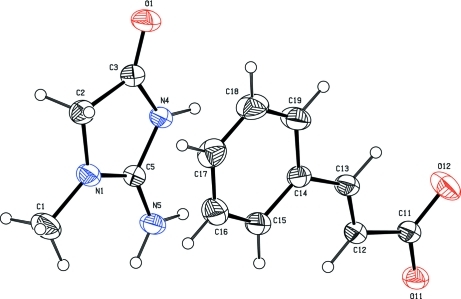
The molecular structure of the title compound with the numbering scheme for the atoms and 50% probability displacement ellipsoids.

**Fig. 2.**
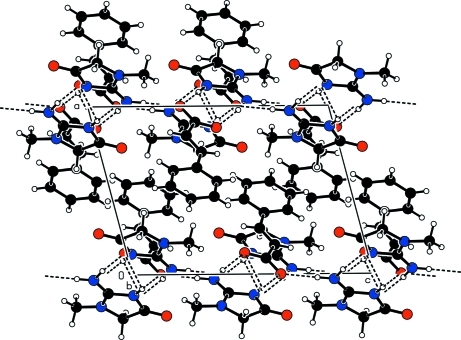
Packing diagram of the molecules viewed down the b axis. Hydrogen bonds are drawn as dashed lines.

**Fig. 3.**
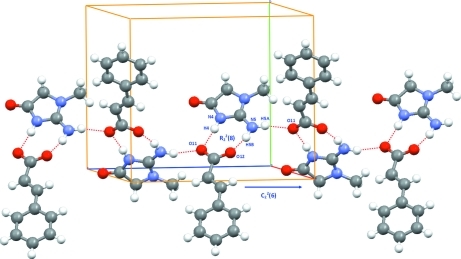
View of ring R22(8) motif and chain C22(6) motifs. Hydrogen bonds are drawn as dashed lines.

## supplementary crystallographic information

### Comment

Noncovalent interactions play a vital role in crystal engineering and supramolecular chemistry. Their strength and directionality is responsible for crystal packing and entire molecular arrays (Desiraju, 1989). Our interest lies in the specificity of recognition between inorganic/organic acids and creatinine. Creatinine is a blood metabolite of considerable importance in clinical chemistry, particularly as an indicator of renal function. It has been proven that determination of creatinine is more valuable for the detection of renal dysfunction than that of urea (Sharma *et al.*, 2004). In renal physiology, creatinine clearance (Madaras & Buck, 1996) is the volume of blood plasma that is cleared of creatinine per unit time. Clinically, creatinine clearance is a useful measure for estimating the glomerular filtration rate of the kidneys. An abnormal level of creatinine in biological fluids is an indicator of various diseases (Narayanan & Appleton, 1980).

The asymmetric part of the title compound contains one creatininium cation and one cinnamate anion (Fig. 1). The protonation of the N site of the cation is evident from C—N bond distances. The values are comaparable with creatininium oxalate monohydrate (Bahadur, Kannan *et al.*, 2007), creatininium benzoate (Bahadur, Sivapragasam *et al.*, 2007) and bis(creatininium) sulfate (Bahadur, Rajalakshmi *et al.*, 2007). The deprotonation on the –COOH group of the cinnamic acid is confirmed from –COO^-^ bond geometry. The planes of the five-membered creatininium ring and the six-membered cinnamate ring are oriented almost parallel to each other with the dihedral angle of 4.5 (1)°. The plane of the deprotonated carboxylate group is twisted out from the plane of aromatic ring by an angle of 11.5 (3)°.

The crystal structure is stabilized by N—H···O hydrogen bonds (Fig. 2; Table 1). Cations are linked to anions forming ion pairs through two N—H···O bonds that produce ring *R*_2_^2^(8) motifs around inversion centres (Bernstein *et al.*, 1995). These ionic dimers are planar and stacked with a dihedral angle of 74.9 (3)°. Further, these adjacent dimers are connected *via* another N—H···O hydrogen bond leading to chain C_2_^2^(6) motif extending along *b* axis of the unit cell (Fig. 3). Alternate hydrophilic and hydrophobic regions are observed along the *a* axis of the unit cell. The hydrophobic regions are located  at *x*~1/2 whereas the hydrophilic regions are located between the hydrophilic layers at *x*~0.

### Experimental

The title compound was crystallized from an aqueous mixture containing creatinine and cinnamic acid in the stoichiometric ratio of 1:1 at room temperature by slow evaporation technique.

### Refinement

All the H atoms except the atoms involved in hydrogen bonds were positioned geometrically and refined using a riding model, with C—H = 0.93 (–CH) and 0.96 Å (–CH_3_) and *U*_iso_(H) = 1.2–1.5 *U*_eq_ (parent atom). H atoms involved in hydrogen bonds were located from differential Fourier maps and refined isotropically.

### Crystal data

**Table:**

C_4_H_8_N_3_O^+^·C_9_H_7_O_2_^−^	*F*(000) = 552
*M**_r_* = 261.28	*D*_x_ = 1.357 Mg m^−^^3^
Monoclinic, *P*2_1_/*c*	Mo *K*α radiation, λ = 0.71073 Å
Hall symbol: -P 2ybc	Cell parameters from 3849 reflections
*a* = 9.1680 (8) Å	θ = 2.1–24.5°
*b* = 11.3391 (11) Å	µ = 0.10 mm^−^^1^
*c* = 12.7070 (12) Å	*T* = 293 K
β = 104.578 (2)°	Block, colourless
*V* = 1278.5 (2) Å^3^	0.25 × 0.22 × 0.18 mm
*Z* = 4	

### Data collection

**Table:**

Bruker SMART APEX CCD area-detector diffractometer	2037 reflections with *I* > 2σ(*I*)
Radiation source: fine-focus sealed tube	*R*_int_ = 0.023
graphite	θ_max_ = 25.0°, θ_min_ = 2.3°
ω scans	*h* = −10→10
9014 measured reflections	*k* = −13→13
2250 independent reflections	*l* = −15→15

### Refinement

**Table:**

Refinement on *F*^2^	Secondary atom site location: difference Fourier map
Least-squares matrix: full	Hydrogen site location: inferred from neighbouring sites
*R*[*F*^2^ > 2σ(*F*^2^)] = 0.035	H atoms treated by a mixture of independent and constrained refinement
*wR*(*F*^2^) = 0.100	*w* = 1/[σ^2^(*F*_o_^2^) + (0.0546*P*)^2^ + 0.2262*P*] where *P* = (*F*_o_^2^ + 2*F*_c_^2^)/3
*S* = 1.05	(Δ/σ)_max_ < 0.001
2250 reflections	Δρ_max_ = 0.16 e Å^−^^3^
186 parameters	Δρ_min_ = −0.14 e Å^−^^3^
0 restraints	Extinction correction: *SHELXL97* (Sheldrick, 2008), Fc^*^=kFc[1+0.001xFc^2^λ^3^/sin(2θ)]^-1/4^
Primary atom site location: structure-invariant direct methods	Extinction coefficient: 0.047 (4)

### Special details

**Table:**

Geometry. All e.s.d.'s (except the e.s.d. in the dihedral angle between two l.s. planes) are estimated using the full covariance matrix. The cell e.s.d.'s are taken into account individually in the estimation of e.s.d.'s in distances, angles and torsion angles; correlations between e.s.d.'s in cell parameters are only used when they are defined by crystal symmetry. An approximate (isotropic) treatment of cell e.s.d.'s is used for estimating e.s.d.'s involving l.s. planes.
Refinement. Refinement of *F*^2^ against ALL reflections. The weighted *R*-factor *wR* and goodness of fit *S* are based on *F*^2^, conventional *R*-factors *R* are based on *F*, with *F* set to zero for negative *F*^2^. The threshold expression of *F*^2^ > σ(*F*^2^) is used only for calculating *R*-factors(gt) *etc*. and is not relevant to the choice of reflections for refinement. *R*-factors based on *F*^2^ are statistically about twice as large as those based on *F*, and *R*- factors based on ALL data will be even larger.

### Fractional atomic coordinates and isotropic or equivalent isotropic displacement parameters (Å^2^)

**Table:**

	*x*	*y*	*z*	*U*_iso_*/*U*_eq_	
C11	0.90518 (15)	−0.14215 (12)	0.42786 (10)	0.0444 (3)	
C12	0.82555 (14)	−0.03500 (11)	0.37734 (10)	0.0425 (3)	
H12	0.8449	−0.0046	0.3142	0.051*	
C13	0.72755 (15)	0.01743 (12)	0.42137 (10)	0.0465 (3)	
H13	0.7159	−0.0168	0.4853	0.056*	
C14	0.63452 (14)	0.12096 (11)	0.38527 (10)	0.0431 (3)	
C15	0.61909 (16)	0.17391 (12)	0.28495 (11)	0.0501 (3)	
H15	0.6716	0.1446	0.2368	0.060*	
C16	0.52597 (19)	0.26999 (14)	0.25656 (13)	0.0620 (4)	
H16	0.5149	0.3046	0.1887	0.074*	
C17	0.44942 (18)	0.31527 (14)	0.32702 (14)	0.0627 (4)	
H17	0.3885	0.3813	0.3076	0.075*	
C18	0.46263 (19)	0.26350 (16)	0.42554 (14)	0.0685 (5)	
H18	0.4099	0.2934	0.4733	0.082*	
C19	0.55385 (19)	0.16718 (15)	0.45395 (12)	0.0616 (4)	
H19	0.5618	0.1319	0.5212	0.074*	
C5	0.88984 (14)	0.38873 (11)	0.37801 (10)	0.0404 (3)	
C3	0.78741 (16)	0.45605 (12)	0.50957 (11)	0.0490 (3)	
C2	0.74062 (17)	0.53562 (12)	0.41229 (11)	0.0514 (4)	
H2A	0.6319	0.5368	0.3846	0.062*	
H2B	0.7763	0.6155	0.4299	0.062*	
C1	0.8109 (2)	0.53413 (16)	0.23096 (13)	0.0705 (5)	
H1A	0.8739	0.6030	0.2417	0.106*	
H1B	0.7096	0.5559	0.1944	0.106*	
H1C	0.8481	0.4779	0.1875	0.106*	
N1	0.81251 (13)	0.48238 (10)	0.33492 (9)	0.0477 (3)	
N4	0.87699 (13)	0.37178 (10)	0.48180 (9)	0.0445 (3)	
N5	0.96941 (14)	0.32061 (11)	0.33272 (10)	0.0490 (3)	
O11	0.99961 (12)	−0.18945 (8)	0.38395 (8)	0.0530 (3)	
O12	0.87297 (14)	−0.18107 (10)	0.51056 (9)	0.0711 (4)	
O1	0.75459 (15)	0.46421 (10)	0.59527 (9)	0.0710 (4)	
H4	0.9161 (18)	0.3101 (15)	0.5239 (14)	0.060 (5)*	
H5A	0.9778 (18)	0.3305 (14)	0.2643 (15)	0.058 (4)*	
H5B	1.0243 (19)	0.2638 (16)	0.3789 (15)	0.066 (5)*	

### Atomic displacement parameters (Å^2^)

**Table:**

	*U*^11^	*U*^22^	*U*^33^	*U*^12^	*U*^13^	*U*^23^
C11	0.0542 (7)	0.0438 (7)	0.0369 (6)	0.0026 (6)	0.0146 (5)	0.0020 (5)
C12	0.0507 (7)	0.0421 (7)	0.0361 (6)	0.0009 (5)	0.0134 (5)	0.0027 (5)
C13	0.0584 (8)	0.0485 (7)	0.0338 (6)	0.0048 (6)	0.0137 (6)	0.0018 (5)
C14	0.0480 (7)	0.0435 (7)	0.0385 (7)	0.0017 (5)	0.0125 (5)	−0.0033 (5)
C15	0.0614 (8)	0.0500 (8)	0.0425 (7)	0.0052 (6)	0.0196 (6)	−0.0004 (6)
C16	0.0775 (10)	0.0569 (9)	0.0514 (8)	0.0108 (8)	0.0155 (7)	0.0125 (7)
C17	0.0643 (9)	0.0541 (9)	0.0671 (10)	0.0176 (7)	0.0116 (8)	0.0003 (7)
C18	0.0749 (10)	0.0713 (11)	0.0660 (10)	0.0226 (9)	0.0301 (8)	−0.0061 (8)
C19	0.0767 (10)	0.0682 (10)	0.0463 (8)	0.0190 (8)	0.0276 (7)	0.0051 (7)
C5	0.0469 (7)	0.0404 (7)	0.0353 (6)	−0.0022 (5)	0.0130 (5)	0.0000 (5)
C3	0.0625 (8)	0.0446 (7)	0.0451 (7)	0.0008 (6)	0.0231 (6)	−0.0030 (6)
C2	0.0596 (8)	0.0469 (8)	0.0507 (8)	0.0092 (6)	0.0196 (6)	0.0002 (6)
C1	0.0960 (12)	0.0713 (11)	0.0481 (9)	0.0244 (9)	0.0257 (8)	0.0208 (7)
N1	0.0600 (7)	0.0469 (6)	0.0385 (6)	0.0083 (5)	0.0165 (5)	0.0061 (5)
N4	0.0596 (7)	0.0415 (6)	0.0363 (6)	0.0050 (5)	0.0194 (5)	0.0036 (5)
N5	0.0634 (7)	0.0509 (7)	0.0374 (6)	0.0112 (6)	0.0216 (5)	0.0045 (5)
O11	0.0690 (6)	0.0500 (6)	0.0451 (5)	0.0160 (4)	0.0240 (5)	0.0054 (4)
O12	0.0861 (8)	0.0757 (8)	0.0637 (7)	0.0326 (6)	0.0415 (6)	0.0330 (6)
O1	0.1074 (9)	0.0644 (7)	0.0550 (6)	0.0145 (6)	0.0460 (6)	−0.0001 (5)

### Geometric parameters (Å, °)

**Table:**

C11—O12	1.2419 (16)	C19—H19	0.9300
C11—O11	1.2616 (16)	C5—N5	1.2928 (17)
C11—C12	1.4784 (18)	C5—N1	1.3170 (16)
C12—C13	1.3140 (18)	C5—N4	1.3669 (16)
C12—H12	0.9300	C3—O1	1.2041 (16)
C13—C14	1.4558 (19)	C3—N4	1.3631 (17)
C13—H13	0.9300	C2—N1	1.4462 (16)
C14—C19	1.3814 (18)	C2—H2A	0.9700
C14—C15	1.3836 (19)	C2—H2B	0.9700
C15—C16	1.375 (2)	C1—N1	1.4422 (17)
C15—H15	0.9300	C1—H1A	0.9600
C16—C17	1.369 (2)	C1—H1B	0.9600
C16—H16	0.9300	C1—H1C	0.9600
C17—C18	1.360 (2)	N4—H4	0.899 (18)
C17—H17	0.9300	N5—H5A	0.899 (18)
C18—C19	1.368 (2)	N5—H5B	0.929 (19)
C18—H18	0.9300		
			
O12—C11—O11	123.98 (12)	N5—C5—N1	126.99 (12)
O12—C11—C12	117.57 (11)	N5—C5—N4	122.74 (12)
O11—C11—C12	118.44 (11)	N1—C5—N4	110.27 (11)
C13—C12—C11	120.18 (12)	O1—C3—N4	126.30 (13)
C13—C12—H12	119.9	O1—C3—C2	127.81 (13)
C11—C12—H12	119.9	N4—C3—C2	105.88 (11)
C12—C13—C14	129.78 (12)	N1—C2—C3	102.94 (11)
C12—C13—H13	115.1	N1—C2—H2A	111.2
C14—C13—H13	115.1	C3—C2—H2A	111.2
C19—C14—C15	118.07 (13)	N1—C2—H2B	111.2
C19—C14—C13	118.11 (12)	C3—C2—H2B	111.2
C15—C14—C13	123.79 (11)	H2A—C2—H2B	109.1
C16—C15—C14	119.89 (13)	N1—C1—H1A	109.5
C16—C15—H15	120.1	N1—C1—H1B	109.5
C14—C15—H15	120.1	H1A—C1—H1B	109.5
C17—C16—C15	120.80 (14)	N1—C1—H1C	109.5
C17—C16—H16	119.6	H1A—C1—H1C	109.5
C15—C16—H16	119.6	H1B—C1—H1C	109.5
C18—C17—C16	119.90 (14)	C5—N1—C1	126.10 (12)
C18—C17—H17	120.0	C5—N1—C2	110.10 (10)
C16—C17—H17	120.0	C1—N1—C2	123.47 (12)
C17—C18—C19	119.63 (14)	C3—N4—C5	110.80 (11)
C17—C18—H18	120.2	C3—N4—H4	124.6 (10)
C19—C18—H18	120.2	C5—N4—H4	124.4 (10)
C18—C19—C14	121.69 (14)	C5—N5—H5A	123.4 (10)
C18—C19—H19	119.2	C5—N5—H5B	114.2 (10)
C14—C19—H19	119.2	H5A—N5—H5B	122.2 (14)
			
O12—C11—C12—C13	1.7 (2)	O1—C3—C2—N1	−179.68 (15)
O11—C11—C12—C13	−179.30 (13)	N4—C3—C2—N1	0.82 (15)
C11—C12—C13—C14	−178.52 (13)	N5—C5—N1—C1	−5.5 (2)
C12—C13—C14—C19	−172.03 (15)	N4—C5—N1—C1	173.73 (14)
C12—C13—C14—C15	9.8 (2)	N5—C5—N1—C2	−179.12 (13)
C19—C14—C15—C16	0.3 (2)	N4—C5—N1—C2	0.16 (15)
C13—C14—C15—C16	178.51 (14)	C3—C2—N1—C5	−0.60 (15)
C14—C15—C16—C17	0.9 (2)	C3—C2—N1—C1	−174.38 (14)
C15—C16—C17—C18	−1.4 (3)	O1—C3—N4—C5	179.71 (15)
C16—C17—C18—C19	0.7 (3)	C2—C3—N4—C5	−0.79 (15)
C17—C18—C19—C14	0.5 (3)	N5—C5—N4—C3	179.74 (12)
C15—C14—C19—C18	−1.0 (2)	N1—C5—N4—C3	0.43 (15)
C13—C14—C19—C18	−179.29 (16)		

### Hydrogen-bond geometry (Å, °)

**Table:**

*D*—H···*A*	*D*—H	H···*A*	*D*···*A*	*D*—H···*A*
N4—H4···O11^i^	0.899 (18)	1.840 (18)	2.7373 (15)	177 (2)
N5—H5A···O11^ii^	0.899 (18)	1.959 (18)	2.8403 (16)	166 (1)
N5—H5B···O12^i^	0.929 (19)	1.754 (19)	2.6663 (16)	167 (2)

Symmetry codes: (i) −*x*+2, −*y*, −*z*+1; (ii) −*x*+2, *y*+1/2, −*z*+1/2.
